# Histological and Macromolecular Characterization of Folliculogenesis in Loggerhead Sea Turtles (*Caretta caretta*): Novel Insights into the Onset of Puberty

**DOI:** 10.3390/ijms26209934

**Published:** 2025-10-12

**Authors:** Ludovica Di Renzo, Erica Trotta, Valentina Notarstefano, Laura Zonta, Elisabetta Giorgini, Luca Marisaldi, Giulia Mariani, Gabriella Di Francesco, Silva Rubini, Marco Matiddi, Cecilia Silvestri, Yakup Kaska, Giulia Chemello, Giorgia Gioacchini

**Affiliations:** 1 Istituto Zooprofilattico Sperimentale dell’Abruzzo e del Molise “G. Caporale”, 64100 Teramo, Italy; l.direnzo@izs.it (L.D.R.); g.mariani@izs.it (G.M.); g.difrancesco@izs.it (G.D.F.); 2 Centro Studi Cetacei Onlus, 65125 Pescara, Italy; 3 Department of Life and Environmental Biology, Università Politecnica delle Marche, Via Brecce Bianche Snc, 60131 Ancona, Italy; e.trotta@pm.univpm.it (E.T.); zontalau@gmail.com (L.Z.); e.giorgini@univpm.it (E.G.); 4 Istituto Nazionale Biostrutture e Biosistemi, Consorzio Interuniversitario (INBB), Via dei Carpegna 19, 00165 Roma, Italy; 5 Faculty of Bioscience and Agro-Food and Environmental Technology, University of Teramo, 64100 Teramo, Italy; vnotarstefano@unite.it; 6 Turtles of the Adriatic Organization APS, Via Tanari 431/A, 40024 Bologna, Italy; luca.marisaldi@taoproject.it; 7 Istituto Zooprofilattico della Lombardia e dell’Emilia-Romagna Sede Territoriale di Ferrara, Via Modena 483, 44124 Ferrara, Italy; silva.rubini@izsler.it; 8 Laboratory of Nekton Ecology (NektonLab.Roma), ISPRA, Italian National Institute for Environmental Protection and Research, Via del Fosso di Fiorano 64, 00143 Rome, Italy; marco.matiddi@isprambiente.it (M.M.); cecilia.silvestri@isprambiente.it (C.S.); 9 Department of Biology, Faculty of Science, Pamukkale University, Denizli 20070, Türkiye; yakupkaska@gmail.com; 10 Sea Turtle Research, Rescue and Rehabilitation Center (DEKAMER), Mugla 48600, Türkiye

**Keywords:** sexual maturity, folliculogenesis, vitellogenesis, histology, FTIR imaging, macromolecular fingerprint, reproductive biology

## Abstract

The Adriatic Sea is a critical neritic habitat for juvenile and adult female loggerhead sea turtles (*Caretta caretta*), where intense anthropogenic pressures and environmental stressors may influence their reproductive biology. Knowledge on the onset of puberty in this population is limited by scarce information on the sub-adult stage, a transitional phase in which reproductive competence is acquired. This study integrated histological analysis and Fourier-transform infrared (FTIR) imaging spectroscopy to provide both structural and biochemical characterization of folliculogenesis, with emphasis on vitellogenesis, in *C. caretta* from the north-central Adriatic Sea. Histological analysis determined the progression of follicle development, while FTIR imaging, a label-free and spatially resolved technique, mapped the distribution of proteins, lipids, and nucleic acids across ovarian compartments. Logistic regression estimated the size at which 50% of females are sexually mature (L_50_) at 58.54 cm Curved Carapace Length (CCL). Based on this value, 60% of sub-adult females were already mature, indicating earlier puberty than previously inferred from macroscopic criteria. These preliminary results, along with reports of sporadic nesting in the Adriatic, raise the question of whether this basin may host further nesting events in the future. FTIR imaging proved to be a powerful tool for reproductive biology in non-model marine vertebrates.

## 1. Introduction

The loggerhead sea turtle (*Caretta caretta*, Linnaeus 1758) is the most abundant sea turtle species in Mediterranean ecosystems, where it plays a key role in nutrient transfer during migrations and provides habitat for epibiont communities [1]. Over the last decades, this ecological niche has been increasingly compromised by anthropogenic activities, including intensive fishing, maritime traffic, and habitat degradation, as well as environmental changes and pollution [2,3,4]. Conservation actions, such as nest monitoring programs and bycatch mitigation measures, have contributed to improving the species’ regional status in the Mediterranean from “vulnerable” to “least concern” [5,6]. Nevertheless, *C. caretta* remains globally classified as “vulnerable,” and important gaps persist in the understanding of its reproductive biology, particularly during the sub-adult stage.

In sea turtles, growth, sexual maturation, and reproduction are influenced by both intrinsic factors, such as physiology, endocrine function, growth rate, and environmental parameters, including temperature, food availability, and contaminant exposure [7,8]. Growth rates differ across populations; for example, Mediterranean Loggerheads typically grow more slowly than their Atlantic counterparts [9,10]. This variation influences the size at which sexual maturity is attained, commonly estimated using Curved Carapace Length (CCL) determined on nesting female. Current classification defines female sea turtle as juveniles < 40 cm CCL, adults > 70 cm CCL, and sub-adults 40–70 cm CCL, representing the intermediate stage [11]. However, this morphological classification is imprecise, as reproductive maturation, marked by the onset of vitellogenesis [12], may precede the first nesting event by several years and can be influenced by other factors that could anticipate or postpone the timing of puberty, such as exposure to endocrine-disrupting chemicals [13,14].

The Adriatic Sea is a site of particular interest, functioning as a critical neritic foraging area for juvenile and adult female loggerhead turtles originating from Greek and Turkish nesting sites in the Aegean Sea [15]. Unfortunately, intensive trawling and other fishing practices in this shallow basin have increased bycatch and stranding rates [16,17], raising concerns about the population’s reproductive potential. Assessing these effects is challenging since the age structure and sexual maturity distribution of loggerhead populations in the Adriatic remain poorly defined [18,19].

The standard approach for determining reproductive maturity, based on histological analysis, allows direct evaluation of gonadal architecture and oocyte developmental stage. However, it does not capture the biochemical processes underlying folliculogenesis and, in particular, vitellogenesis. In this regard, Fourier-Transform Infrared (FTIR) imaging spectroscopy offers a powerful, label-free, and non-destructive technique capable of producing a unique macromolecular fingerprint of biological tissues [20,21,22]. By detecting the vibrational modes of molecular bonds, FTIR imaging enables spatially resolved mapping of proteins, lipids, and nucleic acids within ovarian follicles [23,24]. This high-resolution molecular mapping, when integrated with histological observations, bridges structural and biochemical analyses, offering a more comprehensive understanding of reproductive maturation, especially in non-model species where traditional molecular tools are limited. In this study, histology and FTIR imaging were combined to characterize the structural and molecular changes occurring during folliculogenesis in *C. caretta*, focusing on vitellogenesis. Specifically, this study aims to:

(i) Provide a detailed morphological and biochemical characterization of *C. caretta* folliculogenesis, integrating histological analysis with FTIR imaging spectroscopy;
(ii) Determine, through histological validation, the size at which 50% of females attain sexual maturity (L_50_) in stranded specimens collected along the north-central coast of the Adriatic Sea. This integrative approach provides novel mechanistic insights into the onset of puberty in loggerhead sea turtles and highlights FTIR imaging as a valuable tool for advancing reproductive biology in long-lived marine vertebrates. Although stranded specimens are not fully representative of the entire loggerhead population, the number of dead turtles recovered annually in the study area is considerable, with an estimated 100 individuals [25]. These specimens represent a currently underutilized resource for investigating key aspects of the species’ biology, particularly reproduction.

## 2. Results

### 2.1. Folliculogenesis Development

**Figure 1:**
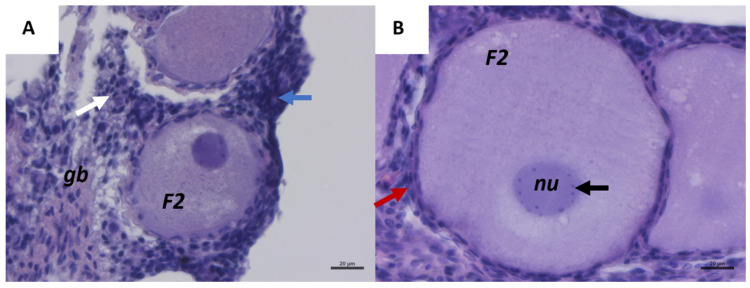
Representative images of *C. caretta* ovary with first and secondary follicle stages, stained with hematoxylin-eosin stain. (**A**) Overview of primordial germinative cells, primordial follicles (white arrow); primary follicles (red arrow); batch of oogonia (blue arrow), and secondary follicle (F2). Scale bar: 20 µm. (**B**) Details of a secondary follicle: bilayer follicular cells (red arrow); nucleolus (black arrow). Scale bar: 20 µm. *gb*, germinative bed; *nu*, nucleus.

Figure 1A represents a histological section of *C. caretta* ovary with a visible germinative bed randomly distributed in the cortex area, which is poorly vascularized and surrounded by connective tissues. In the germinative bed (gb), a batch of oogonia (blue arrow) is visible. Each oogonium has a small and round shape with a central round nucleus and clear cytoplasm. Primordial follicles consist of an oocyte (with a similar oogonia dimension) surrounded by pre-follicular somatic cells (white arrow in Figure 1A). In Figure 1B, a secondary follicle (F2) is shown surrounded by elongated granulosa cells (red arrow), characterized by flat nuclei and arranged in a bilayer structure around the follicle. The nucleus (nu) is central and well-defined with numerous visible nucleoli (black arrow).

**Figure 2:**
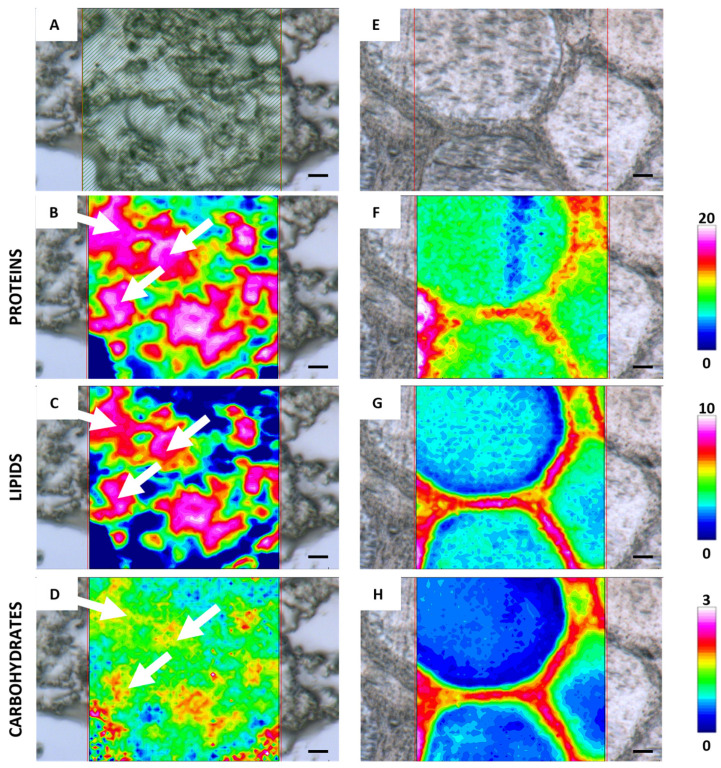
Representative microphotographs of *C. caretta* germinative bed (**A**) and secondary follicle (**E**) and corresponding IR false-color images describing the topographical distribution of: (**B**,**F**) proteins (integration of the IR images under the 1770–1480 cm^−1^ spectral range; scale 0–20); (**C**,**G**) lipids (integration of the IR images under the 3050–2800 cm^−1^ spectral range; scale 0–10), and (**D**,**H**) carbohydrates (integration of the IR images under the 1060–1000 cm^−1^ spectral range; scale 0–3). For all FTIR false-color images, a rainbow color scale was used to represent absorbance intensities (arbitrary units): blue color indicates the lowest values and white the highest ones. Scale bars: 20 µm.

Figure 2 shows the FTIR-imaging analysis of germinative bed and secondary follicles, including the microphotographs (A,E) and the corresponding false color images relative to proteins (B,F), lipids (C,G), and carbohydrates (D,H). The analysis of a batch of oogonia (white arrows) shows a cytoplasmic homogeneous composition in terms of proteins and lipids (Figure 2B,C), the former representing the more abundant macromolecules among those investigated in the germinative bed. The analysis of a secondary follicle evidences that the cytoplasm is still homogeneous in terms of composition and that proteins are still the most abundant macromolecules among those investigated (Figure 2F). Lipids are very poorly concentrated within the oocyte, although they showed higher concentrations corresponding to the membranes of the granulosa cells surrounding the oocyte (Figure 2G). Carbohydrate distribution (Figure 2H) in the inner part of the oocyte (white arrows) reflects the synthesis of cortical alveoli.

**Figure 3:**
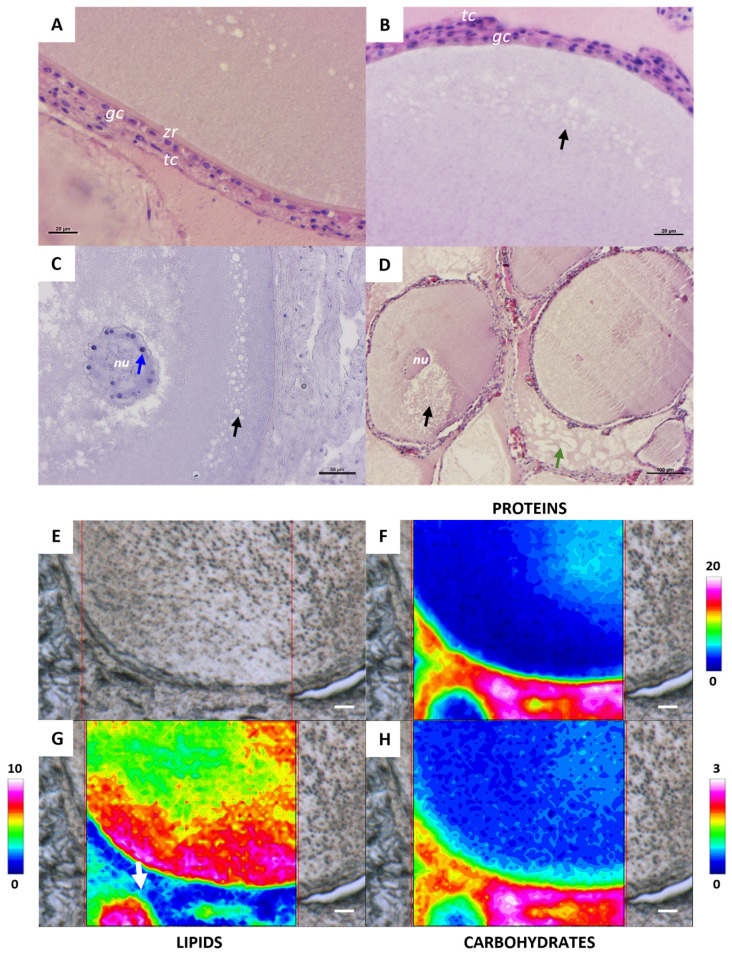
Representative histological images stained with hematoxylin-eosin stain (**A**–**D**) of a *C. caretta* tertiary follicle. (**A**) Details of the zona radiata10 (*zr*) and the follicle bilayer of granulosa cells (*gc*) and theca cells11 (*tc*); scale bar: 20 µm. (**B**) Detail of oil droplets (black arrow); scale bar: 20 µm. (**C**) Details of nucleus (*nu*) and nucleoli (blue arrow); scale bar: 50 µm. (**D**) Panoramic view of tertiary follicles and interstitial fat (green arrow); scale bar: 100 µm. (**E**) Representative microphotograph and corresponding IR false-color images describing the topographical distribution of: (**F**) proteins (integration of the IR images under the 1770–1480 cm^−1^ spectral range; scale 0–20); (**G**) lipids (integration of the IR images under the 3050–2800 cm^−1^ spectral range; scale 0–10), and (**H**) carbohydrates (integration of the IR images under the 1060–1000 cm^−1^ spectral range; scale 0–3). For all FTIR false-color images, a rainbow color scale was used to represent absorbance intensities (arbitrary units): blue color indicates the lowest values and white the highest ones. (**E**–**H**) scale bars: 20 µm.

In the tertiary follicle stage, the zona radiata is more evident, although it is not completely structured (Figure 3A). The analysis of the topographical distribution of proteins and carbohydrates highlights that the zona radiata is composed of glycosylated proteins (Figure 3F,H). The zona radiata is externally surrounded by two layers of theca cells, enclosed by a bilayer of cuboidal granulosa cells (Figure 3A,B). At the beginning of this stage, oil droplets are visible in the cortical area (black arrow) (Figure 3B–D), and their presence was also confirmed by FTIR analysis (Figure 3G). The nucleus maintains the morphology and position observed in the previous stage, and nucleoli are still clearly visible (blue arrow) (Figure 3C). The cytoplasm, excluding the cortical area where oil droplets are located, appears homogeneous, as demonstrated by both histological (Figure 3D) and macromolecular analyses, showing a uniform distribution of proteins and carbohydrates (Figure 3F,H). In the interstitial area between follicles is evident the presence of small fat accumulation (green arrow and withe arrow, Figure 3D,G, respectively).

**Figure 4:**
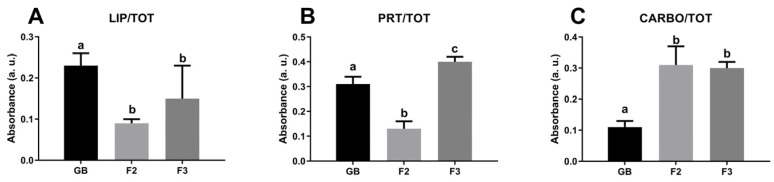
Macromolecular composition of the cytoplasm of the germinative bed (GB), secondary follicle (F2), and tertiary follicle (F3) of *C. caretta*. Univariate analysis of band area ratios representative of relative amounts of: (**A**) lipids (LIP/TOT), (**B**) proteins (PRT/TOT), and (**C**) carbohydrates (CARBO/TOT). Different letters indicate statistically significant differences among experimental groups.

A semiquantitative analysis of spectral data was also performed to characterize the cytoplasm of germinative bed (GB), secondary (F2), and tertiary (F3) oocytes (Figure 4). Among the three different stages analyzed, the cytoplasm of secondary oocytes contains significant lowest levels of lipids if compared to GB and FE (*p* < 0.0001, *p* < 0.05, respectively), while oocytes from the germinative bed are the richest in lipids (LIP/TOT; Figure 4A). Concerning proteins amount, tertiary oocytes show the highest levels compared to GB and F2 (*p* < 0.0001 both), the last one has the lowest protein amount observed (PRT/TOT; Figure 4B). Finally, the germinative bed is characterized by oocytes with the lowest carbohydrate content compared to F2 and F3 (*p*< 0.0001 both) (CARBO/TOT; Figure 4C).

**Figure 5:**
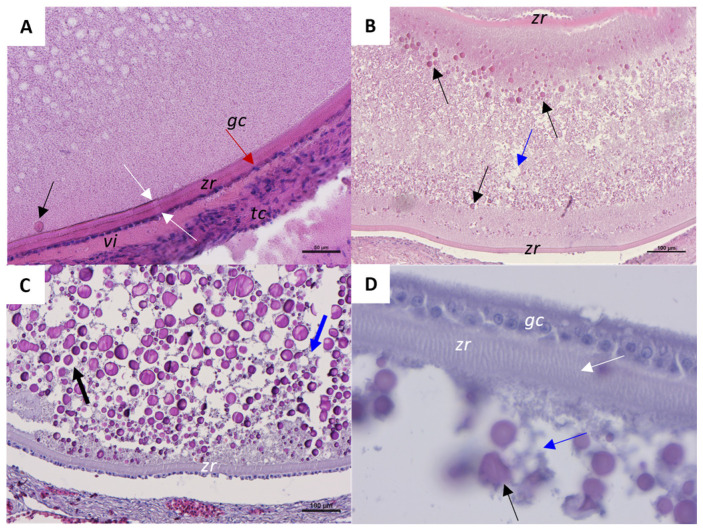
Representative histological images of a *C. caretta* vitellogenic follicle stained with hematoxylin-eosin stain. (**A**) beginning of vitellogenesis; scale bar: 50 µm. (**B**) mid phase of vitellogenesis; scale bar: 100 µm. (**C**) Details of yolk vesicles and oil droplets distribution at the late stage of vitellogenesis; scale bar: 100 µm. (**D**) Detail of zona radiata; Scale bar: 20 µm. *tc*, theca cells; *zr*, zona radiata; *gc*, granulosa cells (red arrow); *vi*, vitellogenin; white arrow, inner and outer layer of zona radiata; blue arrow, oil droplets; black arrow, yolk vesicle.

At the beginning of early vitellogenesis (Figure 5A), the follicle is characterized by elongated and multilayered theca cells (*tc*) and a single layer of granulosa cells (*gc*) (red arrow). The outer and inner layers of the zona radiata (*zr*) are distinguishable (white arrows in Figure 5A). Between the *tc* layers and the *gc* layer, an initial accumulation of vitellogenin (*vi*) is evident. In the mid phase of vitellogenesis, the first yolk vesicles are visible in the cortical region of the oocyte (black arrows in Figure 5B). Also, oil droplets are now numerous and distributed in the entire cytoplasmic area (blue arrow in Figure 5B). Once the follicle reaches the late vitellogenic stage (Figure 5C), the ooplasm is mainly occupied by the spherical yolk vesicles (black arrow) and oil droplets (blue arrow). At the late phase of vitellogenesis, the thick and differentiated zona radiata (*zr*) is characterized by many cytoplasmic bridges (white arrow) connecting the granulosa cells with the ooplasm (Figure 5D).

**Figure 6:**
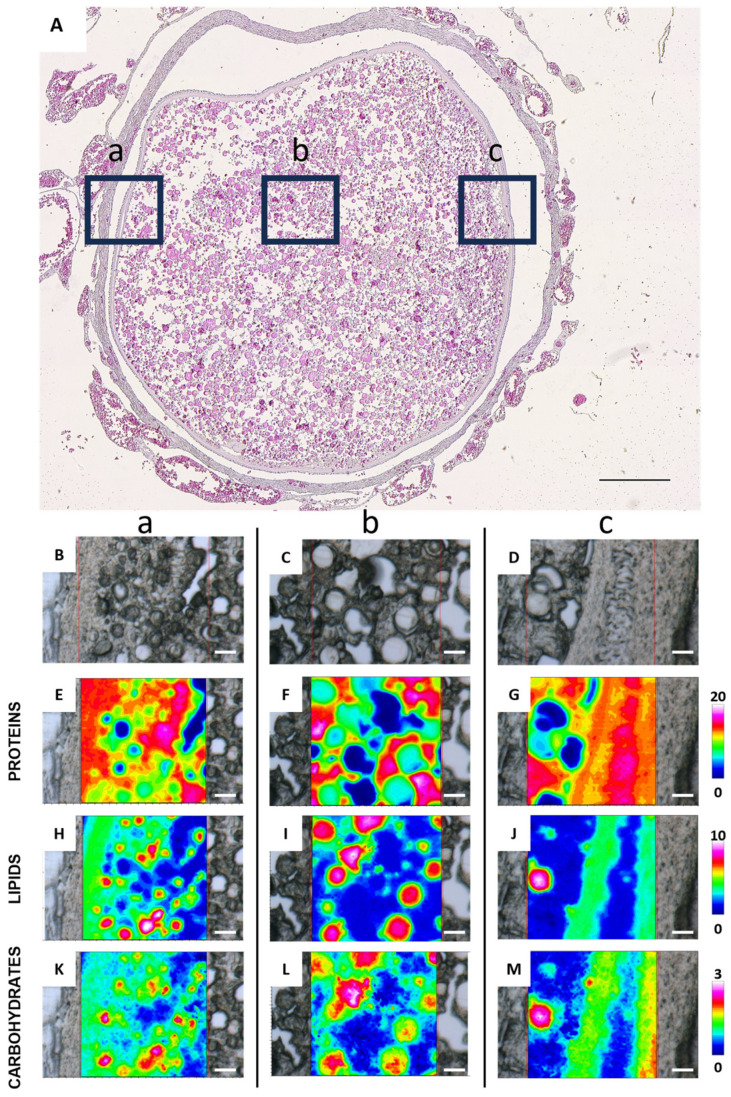
Representative histological and FTIR imaging analysis of a *C. caretta* late vitellogenic follicle. (**A**) Histological overview of the follicle, with three selected regions marked by black squares: (**a**) outer left, (**b**) inner, and (**c**) outer right areas. Scale bar: 200 µm. (**B**–**D**) Representative microphotographs of the above defined areas, and corresponding IR false-color images describing the topographical distribution of: (**E**–**G**) proteins (integration of the IR images under the 1770–1480 cm^−1^ spectral range; scale 0–20); (**H**–**J**) lipids (integration of the IR images under the 3050–2800 cm^−1^ spectral range; scale 0–10), and (**K**–**M**) carbohydrates (integration of the IR images under the 1060–1000 cm^−1^ spectral range; scale 0–3). For all FTIR false-color images, a rainbow color scale was used to represent absorbance intensities (arbitrary units): blue color indicates the lowest values and white the highest ones. (**B**–**M**) scale bars: 20 µm.

**Figure 7:**
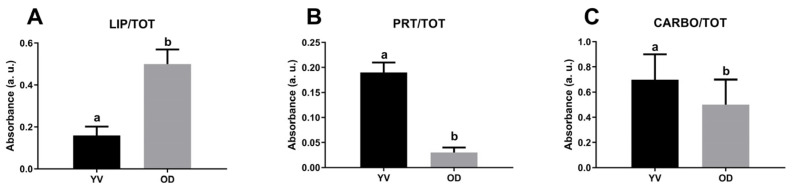
Macromolecular composition of yolk vesicles (YV) and oil droplets (OD) in vitellogenic oocytes of *C. caretta*. Univariate analysis of band area ratios representative of relative amounts of: (**A**) lipids (LIP/TOT), (**B**) proteins (PRT/TOT), and (**C**) carbohydrates (CARBO/TOT). Different letters indicate statistically significant differences among experimental groups.

Figure 6 shows the FTIR imaging analysis of three different areas of a late vitellogenic follicle, focusing on the outer layers (points a and c) and the interior of the oocyte (point b) (Figure 6A). The vibrational analysis at points a and c reveals the presence of endosome entry involving two types of vesicles: one containing more proteins (Figure 6E,G) and the other one containing lipids (Figure 6H,J) and carbohydrates (Figure 6K,M). Protein distribution in Figure 6G indicates cytoplasmic bridges connecting granulosa cells with the oocyte cytoplasm. The analysis of the topographical distribution of major macromolecules of the area c related to the internal part of the oocyte, shown in Figure 6F,I,L, confirms the presence of two distinct vesicle types representing yolk components: one richer in lipids and carbohydrates, and the other richer in proteins. The macromolecular characterization of yolk vesicles (YV) and oil droplets (OD) in vitellogenic oocytes was also obtained by a semiquantitative analysis of the spectral data (Figure 7). Oil droplets contain lipids above all (LIP/TOT, *p* < 0.0001, Figure 7A), while yolk vesicles (YV) appear rich in proteins (PRO/TOT, *p* < 0.0001, Figure 7B). As for carbohydrates, their content is similar in both the yolk vesicles and the oil droplets (CARBO/TOT, *p* < 0.001, Figure 7C).

**Figure 8:**
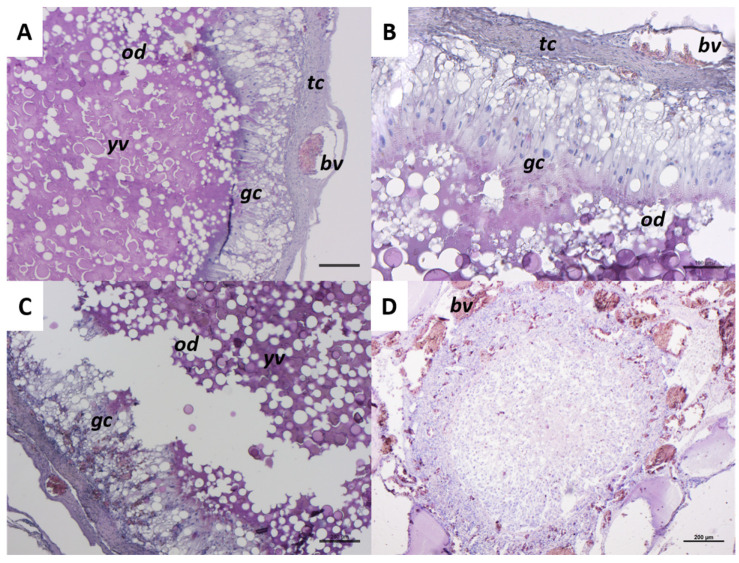
Representative images of a *C. caretta* follicle atretic progression stained with hematoxylin-eosin stain. (**A**–**C**) initial phase of atresia; scale bars: 100 µm, 100 µm, and 200 µm, respectively. (**D**) final phase of atresia; scale bar: 200 µm. *gc*, granulosa cells; *tc*, theca cells; *bv*, blood vessel; *od*, oil droplets; *yv*, yolk vesicles.

Figure 8 shows an atretic follicle at the beginning and final phase of the atretic process. The initial phase of follicular atresia (Figure 8A–C) is characterized by granulosa cells (*gc*) that lose their normal cuboidal appearance and shift from a single-layer organization to a 2/4 cell-layers structure (Figure 8A–C). Granulosa cells (*gc*) penetrate inside the follicles, appearing more elongated with clearly visible nuclei, and are identified as ‘giant cells’ (Figure 8B). The zona radiata is no longer visible, while yolk vesicles (*yv*) and oil droplets (*od*) remain evident in the cytoplasm (Figure 8A–C). At this phase, theca cells (*tc*) have a thick layer conformation (Figure 8A,B). The last stage of atresia presents numerous blood vessels (*bv*) in the connective tissue surrounding the follicle. The yolk vesicles are no longer visible in the cytoplasm (Figure 8D).

**Figure 9:**
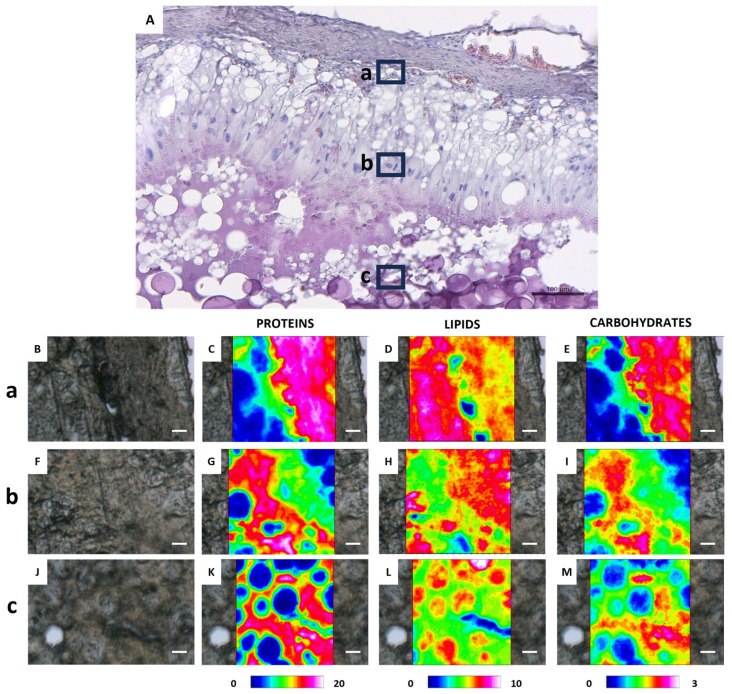
(**A**) Representative histological image (H&E stained) of a *C. caretta* atretic follicle: a, b, and c black squares represent, respectively, the outer, intermediate, and inner areas. Scale bar: 50 µm. (**B**,**F**,**J**) Representative microphotographs of the above defined areas, and corresponding IR false-color images describing the topographical distribution of: (**C**,**G**,**K**) proteins (integration of the IR images under the 1770–1480 cm^−1^ spectral range; scale 0–20); (**D**,**H**,**L**) lipids (integration of the IR images under the 3050–2800 cm^−1^ spectral range; scale 0–10), and (**E**,**I**,**M**) carbohydrates (integration of the IR images under the 1060–1000 cm^−1^ spectral range; scale 0–3). For all FTIR false-color images, a rainbow color scale was used to represent absorbance intensities (arbitrary units): blue color indicates the lowest values and white the highest ones. (**B**–**M**) scale bars: 20 µm.

Figure 9 shows the histological and FTIR-imaging analysis of three different areas (a, b, c) of an atretic follicle at the first stage of the atretic process. Starting from the outer part of the follicle (point a), Figure 9B–E represent the connection between granulosa cells and the outermost thecal cells. FTIR imaging shows that the transfer of macromolecules absorbed by granulosa cells to the thecal cells occurs selectively: proteins and carbohydrates are the first to be transferred, while lipids remain accumulated within the granulosa cells. Figure 9F,I refer to FTIR-imaging of elongated granulosa cells showing that macromolecule uptake by granulosa cells is selective, as lipids continue not to co-localize with proteins and carbohydrates, which instead display the same absorption pattern. While in the inner part, corresponding to point c, Figure 9K shows the remnants of the cytoplasmic content of the atretic oocyte, which is primarily composed of proteins and, to a lesser extent, lipids and carbohydrates (Figure 9L,M), with lipids confined within lipid droplets of various sizes.

**Figure 10:**
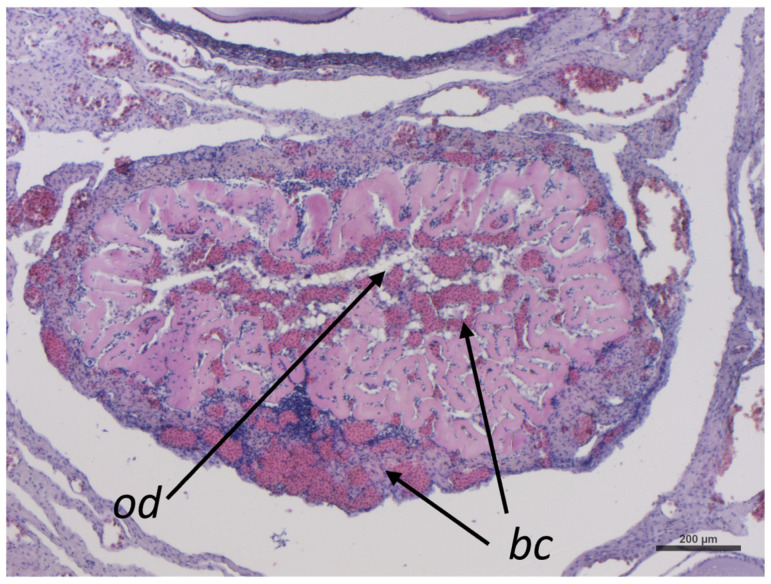
Representative image of *C. caretta* corpus luteum stained with hematoxylin-eosin stain. *bc*, blood cells; *od*, oil droplets. Scale bar: 200 µm.

Post-ovulatory follicles turn into the corpus luteum, which will change into corpus albicans after ceasing progesterone production. The corpus luteum has a compact structure characterized by the aggregation of oil droplets (*od*) and a great accumulation of blood cells (*bc*), and it is also characterized by the collapse of the follicle wall inward (Figure 10).

### 2.2. Histological Sexual Maturity Assessment

**Figure 11:**
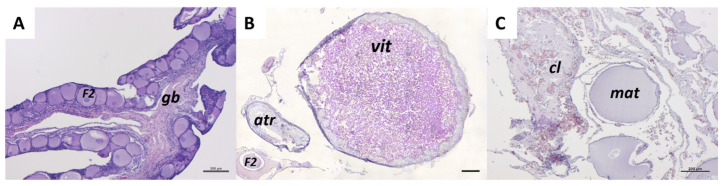
Representative histological images of *C. caretta* ovaries at each reproductive stage stained with hematoxylin-eosin stain. (**A**) immature stage, (**B**) vitellogenic stage, and (**C**) mature stage. *F2*, secondary follicle; *gb*, germinative bed; *vit*, vitellogenic follicle; *atr*, atretic follicles; *mat*, hydrated oocyte; *cl*, corpus luteum. Scale bars: 200 µm.

**Figure 12:**
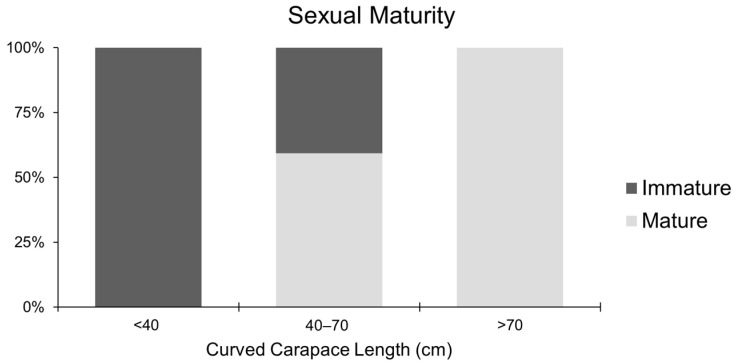
Relative frequency percentage of reproductive stages among each size class: <40; 40–70 and >70.

Based on histological analysis, *C. caretta* females were distinguished into mature and immature according to the evaluation of each reproductive stage (Figure 11). The presence of vitellogenic, mature, atretic, and corpora lutea follicles was interpreted as a marker of sexual maturation. Females at different ovarian maturation stages were grouped into CCL size classes normally used to macroscopically identify juveniles (<40 cm), sub-adults (40–70 cm), and adults (>70 cm). At a size > 40 cm of CCL, 100% of the female gonads analyzed were immature (*n* = 5). In the middle stage between 40 and 70 cm of CCL, the female gonads analyzed were for 60% mature and for 40% immature (*n* = 16 and 11, respectively), while over 70 cm of CCL, only mature female gonads were identified (*n* = 12) (Figure 12). All the examined individuals with a CCL over 60 cm presented mature gonads. Notably, 6% of the analyzed sub-adults presented postovulatory follicles such as corpora lutea and corpora albicans.

### 2.3. L_50_ Analysis

**Figure 13:**
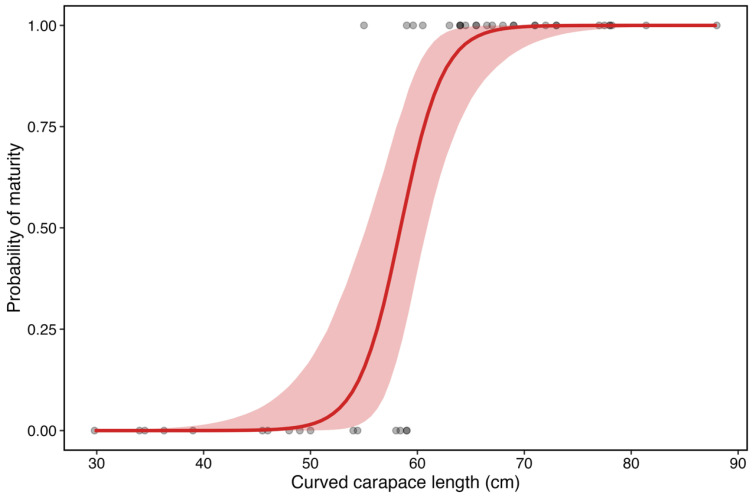
Estimated L_50_ and observed proportion of mature individuals at each 10 cm length class for histology-based classification. The red area corresponds to the 95% credible intervals.

Model diagnostics indicated good convergence (R-hat~1.0, effective sample sizes > 3000), and posterior predictive checks showed that simulated outcomes were consistent with the observed data. The mean posterior predictive probability of maturity across all individuals was 0.6, closely matching the observed proportion of mature individuals (26/44). Maturity probability increased with CCL and posterior slope on standardized CCL was 7.07 (SD = 2.27), corresponding to an odds ratio per cm increase of 1.64 (95% credible interval 1.28–2.43). Thus, the odds of being mature increase by approximately 64% for each additional 1 cm of CCL. Posterior L50 was estimated at 58.54 cm (95% credible interval 55.2–60.9 cm), representing the size at which an average individual has a 50% probability of sexual maturity (Figure 13). The 95% prediction interval ranged from 49 to 66.09 cm.

## 3. Discussion

Detailed knowledge of folliculogenesis in *C. caretta* remains limited, with most previous research focusing on general reproductive biology rather than the cellular and molecular mechanisms underlying follicular development. This study provided novel insights by combining histological analysis with FTIR imaging spectroscopy, enabling both morphological and macromolecular characterization of ovarian follicles at different developmental stages. Only one study, presented by Pérez-Bermúdez et al. (2012) [26], performed a comparable analysis assessing the macromolecular composition of follicles in *Eretmochelys imbricata* using histochemistry. The approach employed in the present study extended this work by coupling high-resolution histology with label-free FTIR imaging, enabling spatially resolved biochemical mapping of lipids, proteins, and carbohydrates within *C. caretta* follicles.

FTIR imaging revealed biochemical differences between secondary and tertiary oocytes, with the latter showing higher protein and lipid content. This is consistent with the elevated transcriptional and translational activity in developing oocytes, as evidenced also in other vertebrates by the presence of lampbrush chromosomes and prominent nucleoli [26]. Lipid accumulation reflects hepatic synthesis of neutral fatty acids and their incorporation into the oocyte [26,27,28]. These pre-vitellogenic stages precede hypothalamic–pituitary–gonadal axis activation and are characteristic of immature females [29].

Vitellogenesis marks the onset of sexual maturity and is dependent on gonadotropin stimulation. In oviparous vertebrates, this phase involves the extensive incorporation of yolk, primarily composed of hepatic vitellogenin, a protein–lipid–carbohydrate complex, into the growing oocyte [30,31]. FTIR imaging confirmed that proteins dominate yolk composition in *C. caretta*, with carbohydrates as the least abundant macromolecule, mirroring patterns observed in other reptile eggs [32]. Because yolk reserves are critical for embryonic and early post-hatch development [32,33], the ability of FTIR to quantify and spatially localize these components provides a valuable tool for assessing the oocyte quality. The imaging approach also visualized vitellogenin internalization via endocytosis and demonstrated the spatial segregation of macromolecules within the follicle. Proteins and carbohydrates were preferentially transferred from granulosa to thecal cells, whereas lipids remained largely sequestered in granulosa cells. In atretic follicles, the persistence of residual proteins and carbohydrates in the cytoplasm and lipid accumulation in droplets indicates selective degradation and resource recycling during post-reproductive oocyte resorption. These observations highlighted the capacity of FTIR to reveal fine-scale biochemical dynamics not detectable with conventional histology, offering a baseline for future studies of reproductive impairment in marine reptiles.

The determination of sexual maturity performed through the histological analysis indicates an L_50_ of 58.54 cm CCL, substantially lower than the previous reference from the Mediterranean, corresponding to the size of the smallest nesting females (66.5–84.7 cm CCL) [11,34,35]. This discrepancy highlights the temporal gap between the activation of HPG axis and the occurrence of nesting events. In this study, approximately 60% of sub-adult females (40–70 cm CCL) shows histological evidence of maturity, and 6% bore postovulatory follicles or corpora lutea, indicating recent ovulation and probable nesting. Similar patterns have been reported in Atlantic populations, where vitellogenin has been detected in the blood of morphologically juvenile and sub-adult females [36], underscoring histology’s greater sensitivity compared to morphometrics alone.

Concerning the Adriatic basin, its shallow bathymetry and semi-enclosed nature promote the early recruitment of juveniles to neritic habitats, bypassing the oceanic phase typical of this stage in other regions [37,38,39]. This life-history shift increases the exposure of potentially mature females to local environmental conditions of the shallow Adriatic basin, highly exploited by numerous anthropic activities and susceptible to environmental fluctuations [39,40].

Regional variations in water temperature, prey availability, habitat productivity and contaminant loads are the main factors known to influence growth rates and reproductive investment in reptiles [7,8,36,37,38,39,40,41,42,43,44,45,46,47].

In particular, environmental contaminants such as endocrine-disrupting chemicals, can affect estrogen receptor activity and vitellogenin synthesis [37,43,44], potentially advancing or delaying puberty onset as already observed in different fish species and reptiles [13,14,45,46,47]. The presence of relatively small mature females, in the studied Adriatic area raises concern due to their prolonged exposure to these factors.

Moreover, in recent years, the occurrence of sporadic nesting events in the north-central Adriatic [48], has highlighted the critical issue of post-hatchlings exposure to the Adriatic conditions. If such events are likely to recur, it becomes necessary to further investigate the reproductive biology of this species. Particularly, linking contaminant burdens to reproductive biomarkers could clarify whether anthropogenic pollution influences puberty onset or alters yolk composition, with potential consequences for hatchling viability and recruitment.

Integrating histological and FTIR imaging into stranding network protocols provides an ethically sustainable approach to generate detailed structural and biochemical datasets from dead specimens, avoiding disturbance to live turtles. Although stranded and bycaught individuals may not perfectly represent the overall population [15,16,17,18], they offer a valuable opportunity for monitoring by using biological samples that are usually discarded, yet they represent a critical resource for reproductive biology research. Standardizing sampling protocols across Mediterranean stranding networks would strengthen demographic assessments, support adaptive conservation measures, and ensure that management strategies reflect both biological trends and environmental pressures.

## 4. Materials and Methods

### 4.1. Data and Sample Collection

Forty-four dead female loggerhead sea turtles, stranded or by-caught along the Adriatic coast of Abruzzo, Molise, and Emilia Romagna regions (during the period 2016–2022), were sampled after the evaluation of the conservation status through necroscopies examination by the Istituto Zooprofilattico Sperimentale dell’Abruzzo e del Molise “G. Caporale” (IZSAM) (*n* = 39) and Istituto Zooprofilattico della Lombardia e dell’Emilia Romagna “B. Ubertini” (IZSLER) (*n* = 5) according to an optimized protocol of published guidelines [49,50]. During necropsy, veterinarians of IZSAM and IZSLER assessed the body condition of each turtle according to the classification described by Poppi and Marchiori (2013) [51]. Based on external examination and internal organ preservation, carcasses were classified using a five-code scale: “0: Alive or just died”, “1: Fresh carcass”, “2: Moderate decomposition”, “3: Advanced decomposition”, and “4: Mummified carcass or partial carcass”. Only individuals with code 1, 2, or 3 were considered suitable for histopathological analysis, as suggested in the Poppi and Marchiori, 2013 [51] protocol. Additionally, animals whose cause of death was disease were excluded from the analyses. Only specimens whose death resulted from anthropic interactions, such as bycatch or collisions with boats, were included. For each turtle, the date of carcass collection, conservation status, and biometric data, including CCL and body weight, were recorded. Ovarian samples collected during necropsy were immediately fixed in formaldehyde and stored at 4 °C for a minimum of 24 h.

### 4.2. Histological Analysis

Histological analyses of ovary samples were performed at the Laboratory of Reproduction and Developmental Biology, at the Department of Life and Environmental Science (DiSVA), Polytechnic University of Marche (Ancona, Italy), following the protocol described by Chemello et al., 2023 [52]. Briefly, samples were washed in ethanol 70% three times (15 min each) and stored in the same ethanol solution at 4 °C until the inclusion process. Successively, samples were dehydrated in increasing concentrations of ethanol solutions (80, 95, and 100%), washed with xylene (Bio-Optica, Milano, Italy), and embedded in paraffin (Bio-Optica). Solidified paraffin blocks were cut with a microtome (Leica RM2125 RTS, Nussloch, Germany) to obtain 5 µm sections that were successively stained following Mayer’s hematoxylin and eosin Y (Merck KGaA, Darmstadt, Germany) protocol. Sections were observed using a Zeiss Axio Imager.A2 (Oberkochen, Germany), and images were acquired using a combined color digital camera Axiocam 503 (Zeiss, Oberkochen, Germany). Ovaries were fully sectioned and observed to determine the characteristics of follicles and the gonad maturity stages of each specimen sampled.

### 4.3. FTIR-Imaging Analysis

FTIR-imaging analysis was performed at the Laboratory of Advanced Research Instrumentation (ARI Lab) of the Department of Life and Environmental Sciences, Polytechnic University of Marche (Ancona, Italy), following [20]. Each formalin-preserved ovarian sample was first washed three times using deionized H_2_O, stored at −20 °C. and then, cut by using a cryostat (MC4000, Histo-Line Laboratories, Milan, Italy). From each samples, N.10 adjacent sections (~10 µm thickness) were cut, deposited onto CaF_2_ optical windows (diameter 13 mm, thickness 1 mm) and let air-dry, before FTIR measurements. The instrumental setup included a Bruker INVENIO-R interferometer coupled with a Hyperion 3000 Vis-IR microscope, equipped with a Focal Plane Array (FPA) detector operating at liquid nitrogen temperature (Bruker Optics, Ettlingen, Germany). The photomicrograph of each section was acquired with a 15× objective to accurately select specific areas, corresponding to primary, secondary, tertiary, and vitellogenic follicles, on which IR images were acquired. The acquisition was carried out in transmission mode in the 4000–900 cm^−1^ range, with a spectral resolution of 4 cm^−1^; the FPA detector let acquire 164 × 164-µm squares, consisting of 4096 pixels, with a spatial resolution of 2.56 µm; each pixel corresponded to a spectrum, obtained by accumulating 256 scans. Before the acquisition of each IR image, the background was acquired with the same setup on a clean area of the CaF_2_ optical window and automatically subtracted from subsequent measurements. The following pre-processing procedures were employed for raw IR images on the whole spectral range: Atmospheric Compensation, to eliminate the signals due to the presence of atmospheric carbon dioxide and water vapor, and Vector Normalization, to reduce artifacts caused by possible differences in section thickness and light intensity (OPUS 7.5 software package, Bruker Optics, Ettlingen, Germany). Only IR spectra showing at 1660 cm^−1^ a peak height higher than 0.07 a.u. were considered. No baseline correction was needed. Pre-processed IR images were then used to assess the topographical distribution of macromolecules of interest. To this aim, false-color images were obtained by integrating pre-processed IR images under the 3050–2800 cm^−1^ spectral region (containing the stretching modes of alkyl groups in lipids, LIP), the 1770–1480 cm^−1^ spectral region (containing the vibrational modes of Amide I and II bands of proteins, PRT), and the 1060–1000 cm^−1^ spectral region (containing the stretching modes of C-O-C and C-OH of carbohydrates, CARBO) [20,21,22]. For a deeper analysis of the macromolecular changes in the cytoplasm of the germinative bed (GB), secondary follicle (F2), and tertiary follicle (F3), microareas of ca. 200 IR spectra were chosen. Concomitantly, microareas of ca. 50 IR spectra were chosen on yolk vesicles (YV) and oil droplets (OD) of vitellogenic follicles. These IR spectra were integrated under the spectral regions defined above (Integration routine; OPUS 7.1 software package, Bruker Optics, Ettlingen, Germany). The sum of the integrated areas 3050–2800 and 1770–900 cm^−1^ was considered indicative of the total tissue biomass (TOT). The values of the integrated areas were used to calculate PRT/TOT, LIP/TOT, and CARBO/TOT band area ratios.

### 4.4. Sexual Maturity Evaluation

Sexual maturity was established following the criteria described by Hamann et al. (2003) [29]. Briefly, immature females were identified through the presence in the ovary of oogonia and/or primary and secondary follicles in the germinative bed associated with lipid storage, and the total absence of the vitellogenic process. Females were considered mature when they had ovaries with vitellogenic follicles, corpus luteum, or albicans as distinctive tracts and a restricted stroma. Stained section images were independently evaluated by three blind readers, experienced reproductive biologists with extensive expertise in histological assessment of ovarian development and sexual maturity in marine vertebrates.

### 4.5. Statistical Analysis

The size at 50% maturity (L_50_) is commonly used in other species, such as fishes, to estimate the proportion of mature individuals at a given length or age [53,54,55] and could be an optimal tool to assess the reproductive potential of the loggerhead sea turtle species. The probability of maturity was modeled as a function of carapace length (CCL, cm) using Bayesian logistic regression. This approach requires a binary response variable, therefore sexual maturity was coded as “0” (immature) and “1” (mature) according to the histological assessment of the ovaries from each individual (*n* = 44). The analysis was performed with RStudio (version 2023.1.9.494). [56]. Weakly informative normal priors (mean = 0, SD = 5) on standardized data (mean = 0, SD = 1) were applied to both slope and intercept to stabilize estimates under a small dataset and potential near-complete separation, based on practices recommended by Gelman et al. (2008) [57]. Posterior distributions of the coefficients and odds ratios (OR), together with their 95% credible intervals, and the size at 50% maturity (L_50_) were obtained from 8000 posterior draws. Posterior predictive simulations were used to generate 95% prediction intervals for individual outcomes. The rstanarm package (version 2.32.2) [58] was used for model fitting and ggplot2 [59] for visualization.

FTIR semiquantitative data related to cytoplasm composition of germinative bed, secondary, and tertiary oocytes were analyzed by one-way ANOVA, followed by Tukey’s post-test. FTIR semiquantitative data related to macromolecular composition of yolk vesicles and oil droplets were analyzed by a two-tailed unpaired *t*-test. A Shapiro–Wilk test was performed to assess the normal distribution of the data. The statistical software package Prism5 (GraphPad Software, San Diego, CA, USA) was used; significance was set at *p* < 0.05. All results are presented as mean ± S.D.

## 5. Conclusions

This study provides a comprehensive histological and biochemical characterization of folliculogenesis in *Caretta caretta* from a restricted area of the Adriatic Sea, integrating conventional histological analysis with FTIR imaging spectroscopy. The combined approach enabled the identification of structural and macromolecular changes associated with vitellogenesis and provided a more precise assessment of sexual maturity. The estimated L_50_ of 58.54 cm CCL and the high proportion of sexually mature sub-adult females indicate that puberty may occur earlier than previously inferred from nesting females. These findings, together with recent reports of sporadic nesting events in the studied area, suggests a possible shift in habitat use from an exclusively foraging ground to a potential reproductive area for smaller females. By coupling histology with FTIR imaging, this work demonstrates the utility of high-resolution, label-free molecular mapping for elucidating reproductive processes in long-lived, non-model marine vertebrates. Although authors are conscious of the limitation represented by the relatively small sample size, the approach of this study provides clear evidence for the determination of sexual maturity. This integrative methodology offers valuable opportunities for assessing the reproductive status of threatened populations, informing conservation planning, and identifying potential anthropogenic or environmental factors influencing reproductive physiology. The preliminary results obtained in this study represent the basis for future research that aims to better understand the drivers of *C. caretta* reproductive biology and to refine conservation strategies by extending this approach to other geographic regions.

## Data Availability

The data presented in this study are available on request from the corresponding author due to the utilization of FTIR-imaging metadata for further analysis and publication.
